# Supplementary material for: Large Benefit from Simple Things: High-Dose Vitamin A Improves RBP4-Related Retinal Dystrophy
Source: Int J Mol Sci. 2022 Jun 13;23(12):6590. doi: 10.3390/ijms23126590 (PMC9223508; doi:10.3390/ijms23126590)
Supplement: Supplementary file 1 [file ijms-23-06590-s001.zip › ijms-1716563-supplementary.pdf]

Supplementary Appendix.

Figure S1……………………………………………………p.2
Figure S2……………………………………………………p.3
Figure S3……………………………………………………p.4
Figure S4……………………………………………………p.5

Supplementary Figure S1 . Kinetic visual field

A. Goldman VF before treatment

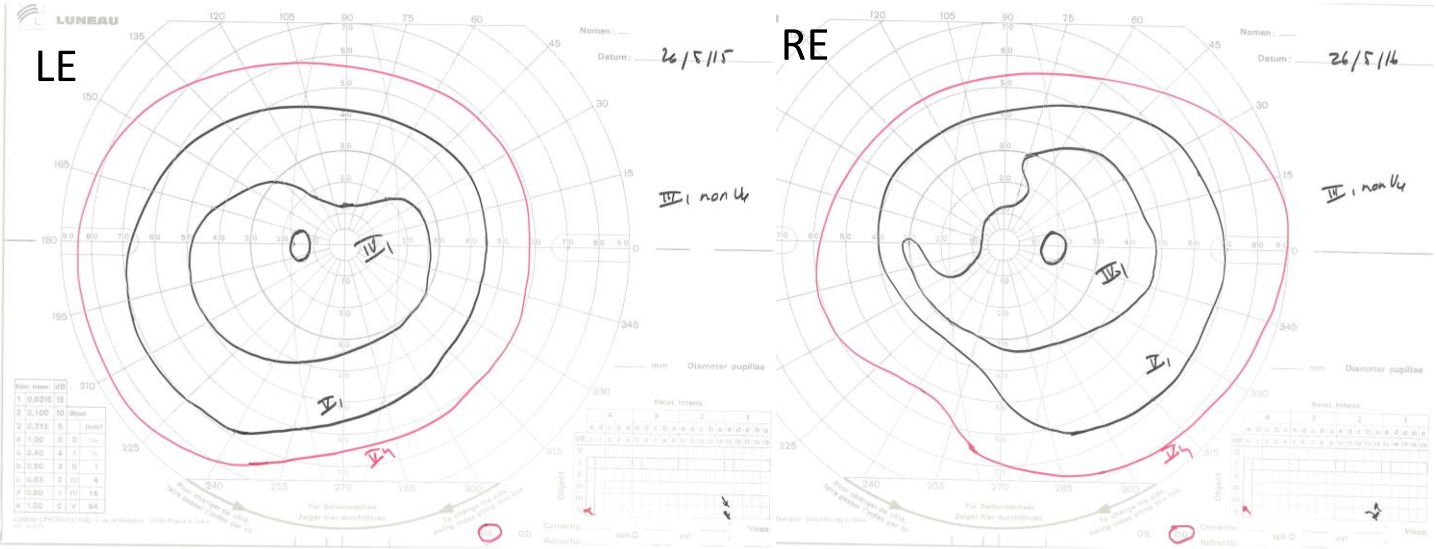

Initial Goldman VF revealed superior defect at IV1e target. III1e and smaller targets have not been perceived.

C. Octopus kinetic VF M+5

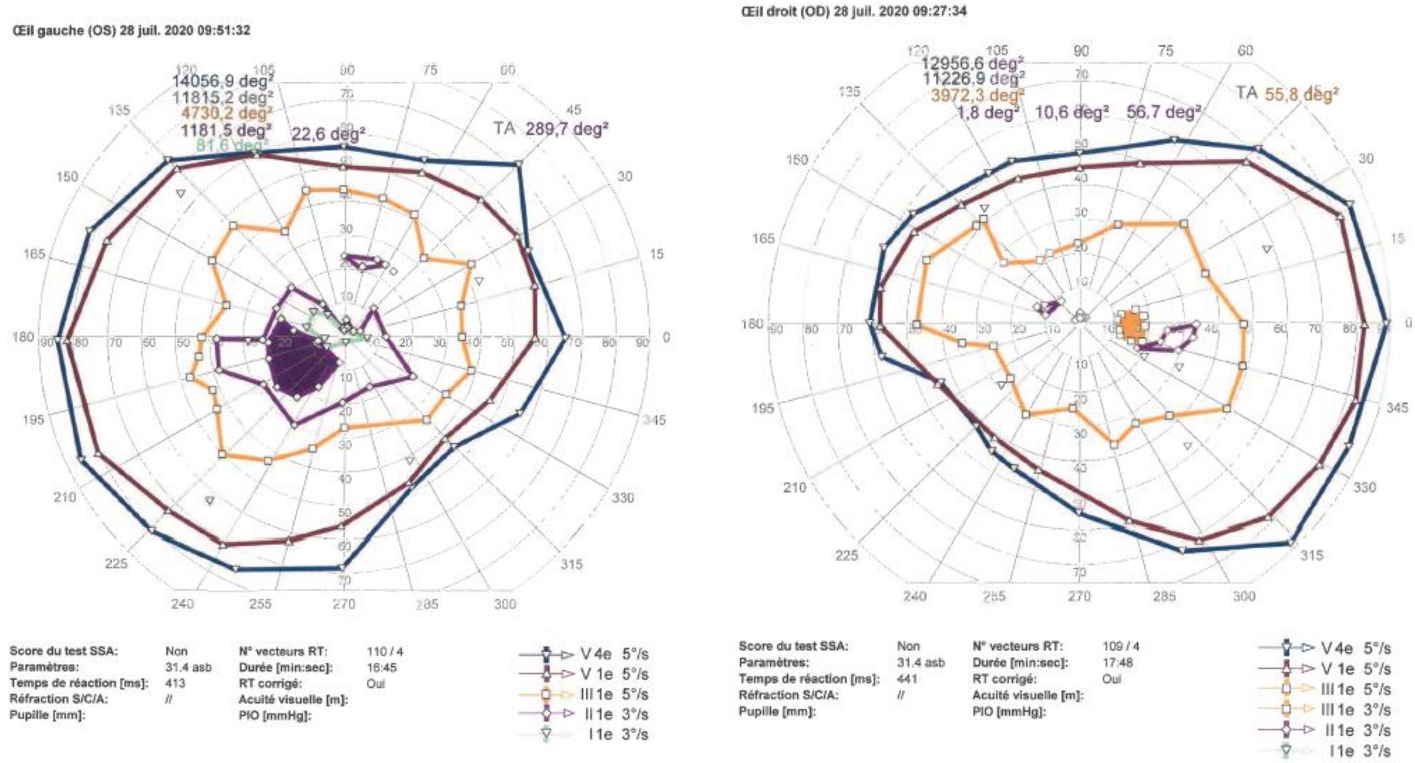

VF changes from baseline at M+5: III1e and II1e targets perceived by both eyes.

C. Octopus kinetic VF M+12

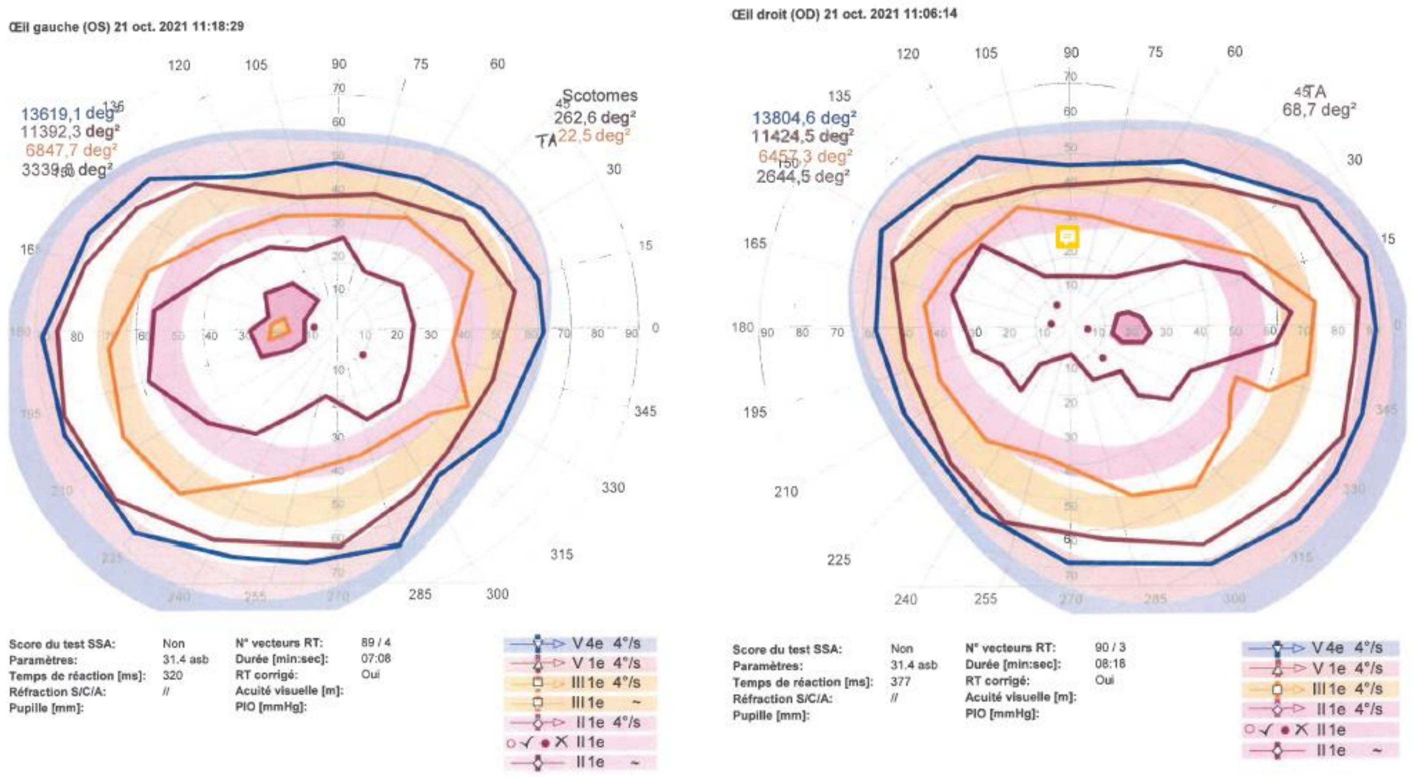

VF changes from baseline at M+12: III1e and II1e isopters enlargement.

Supplementary Figure S2
ffERG

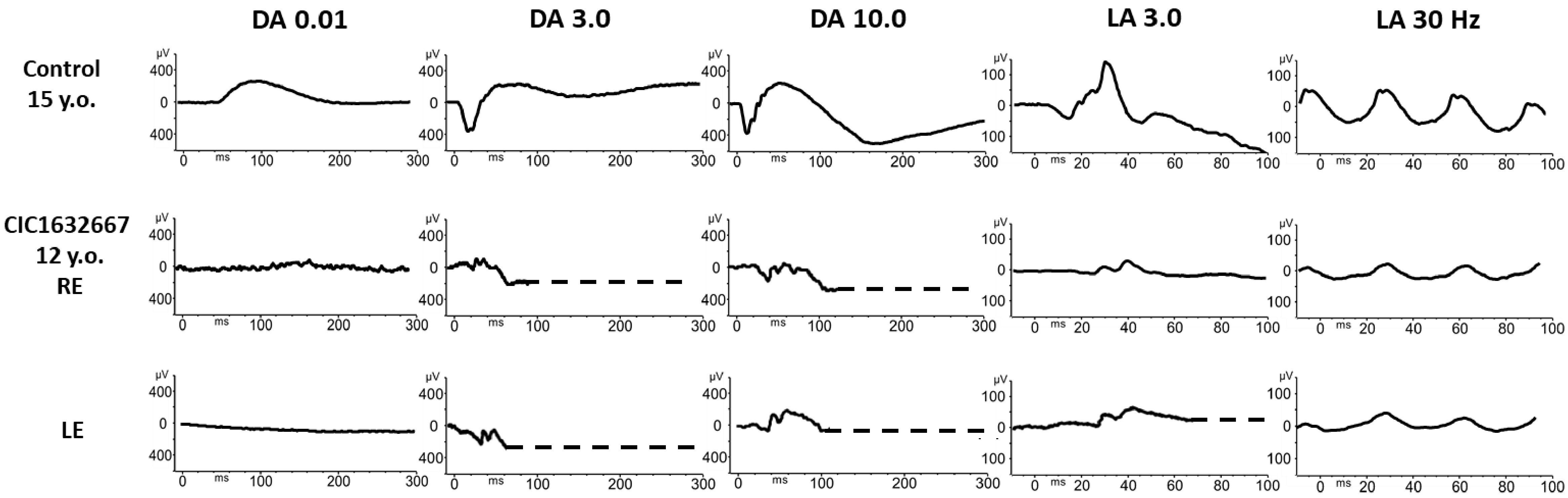

No response to the dim dark adapted flash (DA 0.01), severely reduced amplitude and increased implicit time for the dark adapted bright flashes (DA 3.0 and DA 10.0) and for light-adapted stimuli (LA 3.0 and LA 30 Hz flicker) in keeping with generalized rod-cone dysfunction.

Supplementary Figure S3. Full Stimulus Threshold

A. FST before treatment

Average Results Table												
#	Color	Eye	dB	EB%	EM%	TO	n	Qty	Date	P0	Y	K
4	White	RE	-17,3	0	0	0	3	3	22/10/2020	0	57,942	100

Individual Results Table													
#	Color	Eye	dB	EB%	EM%	TO	n	Qty	Date	P0	Y	K	Time
1	White	RE	-17,3	0	50	0	24	3	22/10/2020	0	57,942	100	00:01:55
2	White	RE	-17,3	0	33	0	32	3	22/10/2020	0	57,942	100	00:02:36
3	White	RE	-17,3	0	50	0	24	3	22/10/2020	0	57,942	100	00:01:55

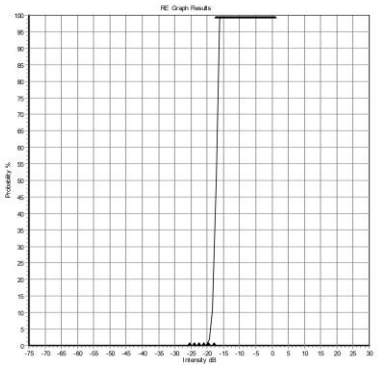

Average Results Table												
#	Color	Eye	dB	EB%	EM%	TO	n	Qty	Date	P0	Y	K
4	White	LE	-15,4	0	0	0	3	3	22/10/2020	0	59,818	100

Individual Results Table													
#	Color	Eye	dB	EB%	EM%	TO	n	Qty	Date	P0	Y	K	Time
1	White	LE	-15,7	0	100	0	32	3	22/10/2020	0	59,547	100	00:02:36
2	White	LE	-15,2	0	100	0	32	3	22/10/2020	0	59,987	100	00:02:36
3	White	LE	-13,6	0	80	0	24	3	22/10/2020	0	62,281	26,534	00:01:55

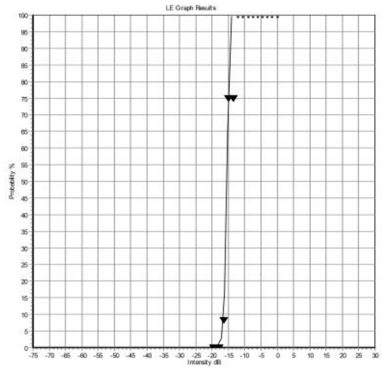

Initial FST to achromatic stimulus revealed severely reduced threshold for the white stimulus under dark-adapted conditions: -17.3dB in RE and -15.4dB in LE

B. FST after treatment M+5

Average Results Table												
#	Color	Eye	dB	EB%	EM%	TO	n	Qty	Date	P0	Y	K
4	White	RE	-21.7	0	0	0	3	3	25/03/2021	0	53,744	49

Individual Results Table													
#	Color	Eye	dB	EB%	EM%	TO	n	Qty	Date	P0	Y	K	Time
1	White	RE	-22,5	0	62	0	32	3	25/03/2021	0	52,867	48,9	00:02:36
2	White	RE	-21,6	0	66	0	32	3	25/03/2021	0	53,552	100	00:02:36
3	White	RE	-21.0	0	66	0	32	3	25/03/2021	0	54,527	37,7	00:02:36

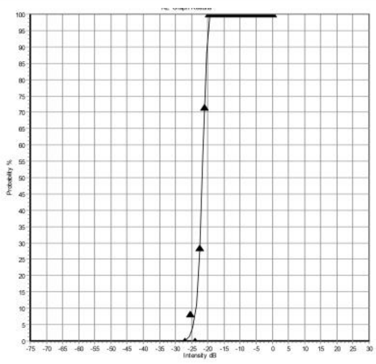

Average Results Table												
#	Color	Eye	dB	EB%	EM%	TO	n	Qty	Date	P0	Y	K
4	White	LE	-21,9	0	0	0	3	3	25/03/2021	0	53,603	36

Individual Results Table													
#	Color	Eye	dB	EB%	EM%	TO	n	Qty	Date	P0	Y	K	Time
1	White	LE	-21,7	0	71	0	32	3	25/03/2021	0	53,513	100	00:02:36
2	White	LE	-21,9	0	57	0	32	3	25/03/2021	0	53,832	25,9	00:02:36
3	White	LE	-22,5	0	0	0	32	3	25/03/2021	0	53,064	31,7	00:02:36

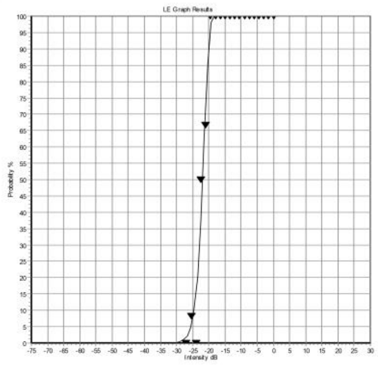

FST changes from baseline at M+5: 25 and 40% dark-adapted threshold decrease for RE and LE respectively

C. FST after treatment M+12

Average Results Table												
#	Color	Eye	dB	EB%	EM%	TO	n	Qty	Date	P0	Y	K
4	White	RE	-38,5	0	0	0	3	2	21/10/2021	0	37,028	27,82

Individual Results Table													
#	Color	Eye	dB	EB%	EM%	TO	n	Qty	Date	P0	Y	K	Time
1	White	RE	-38,8	0	30	0	48	1	21/10/2021	0	36,327	100	00:03:56
2	White	RE	-36,6	0	50	0	40	3	21/10/2021	0	38,53	100	00:03:16
3	White	RE	-39,8	0	40	0	48	1	21/10/2021	0	36,118	14,941	00:03:56

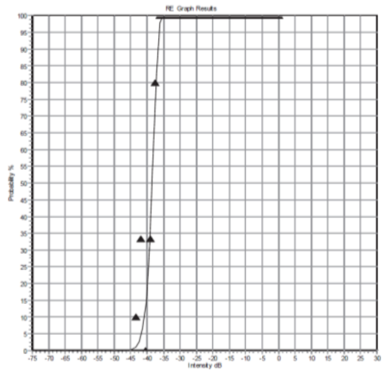

Average Results Table												
#	Color	Eye	dB	EB%	EM%	TO	n	Qty	Date	P0	Y	K
4	White	LE	-48,5	0	0	0	3	1	21/10/2021	0	27,664	8,2438

Individual Results Table													
#	Color	Eye	dB	EB%	EM%	TO	n	Qty	Date	P0	Y	K	Time
1	White	LE	-46,5	0	0	0	56	1	21/10/2021	0	28,605	100	00:04:37
2	White	LE	-47,0	0	10	0	48	1	21/10/2021	0	28,065	100	00:03:56
3	White	LE	-50,9	28	0	0	56	2	21/10/2021	1,008E-010	25,076	9,6285	00:04:37

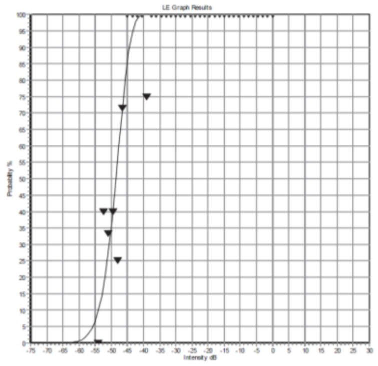

FST changes from baseline at M+12: 123 and 215% dark-adapted threshold decrease for RE and LE respectively

Supplementary Figure S4.
Wide-field fundus photography

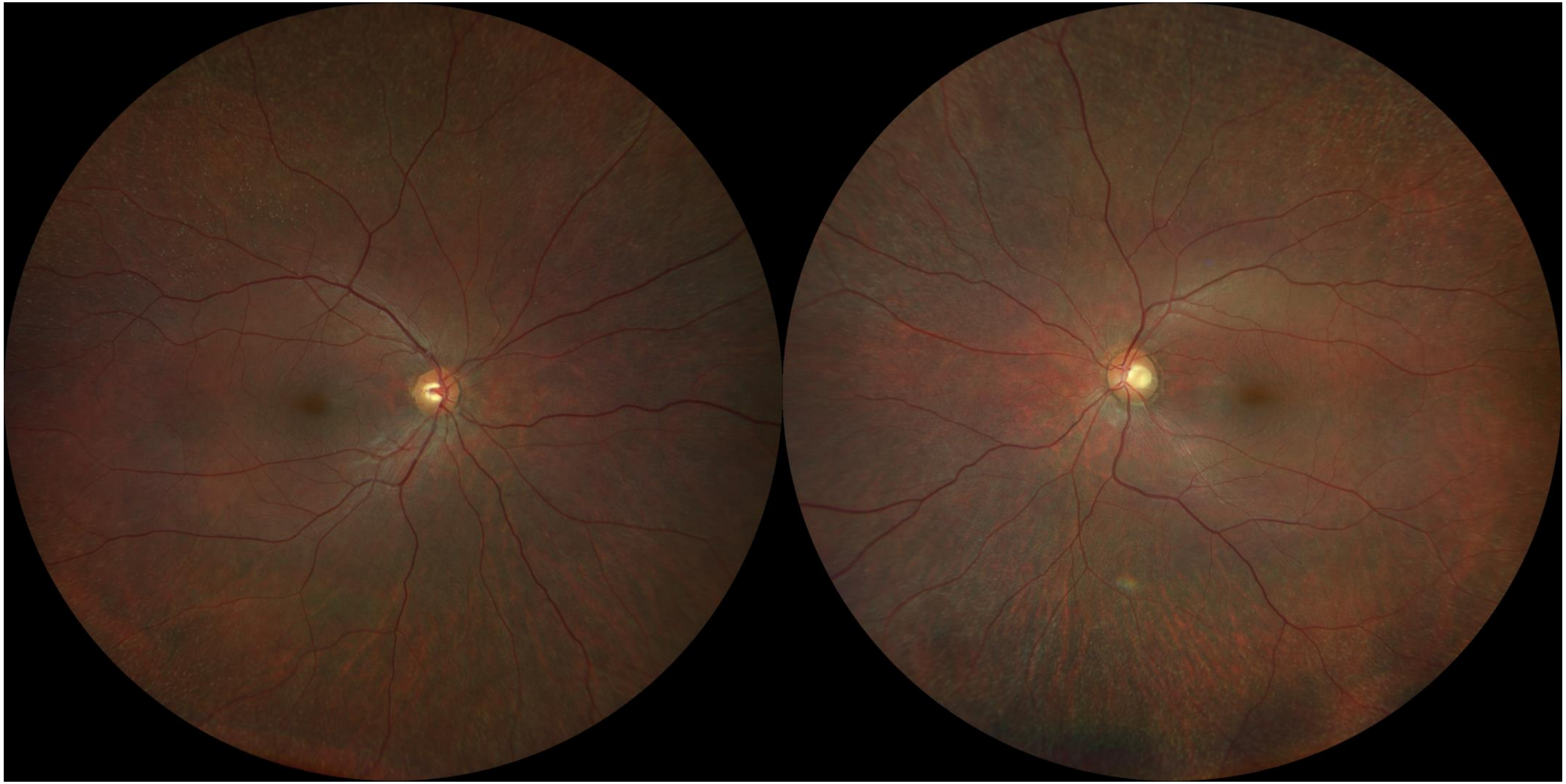

Numerous white dots scattered in the mid and far periphery with no pigmentary or atrophic retinal changes.
